# HNRNPA2B1-mediated m^6^A modification of lncRNA MEG3 facilitates tumorigenesis and metastasis of non-small cell lung cancer by regulating miR-21-5p/PTEN axis

**DOI:** 10.1186/s12967-023-04190-8

**Published:** 2023-06-12

**Authors:** Ke Li, Quan Gong, Xu-Dong Xiang, Gang Guo, Jia Liu, Li Zhao, Jun Li, Nan Chen, Heng Li, Li-Juan Zhang, Chun-Yan Zhou, Zhi-Yong Wang, Li Zhuang

**Affiliations:** 1 Department of Cancer Biotherapy Center, Yunnan Cancer Hospital, the Third Affiliated Hospital of Kunming Medical University, Kunming, 650118 Yunnan China; 2 Department of Rehabilitation and Palliative Medicine, Yunnan Cancer Hospital, the Third Affiliated Hospital of Kunming Medical University, Number 519 Kunzhou Road, Kunming, 650118 Yunnan China; 3 Department of Thoracic Surgery, Yunnan Cancer Hospital, the Third Affiliated Hospital of Kunming Medical University, Kunming, 650118 Yunnan China; 4 Laboratory Zoology Department, Kunming Medical University, Kunming, 650500 Yunnan China; 5 Department of Anesthesiology, Yunnan Cancer Hospital, the Third Affiliated Hospital of Kunming Medical University, Kunming, 650118 Yunnan China

**Keywords:** m^6^A, HNRNPA2B1, lncRNA MEG3, miR-21-5p, NSCLC, Proliferation

## Abstract

**Background:**

Accumulating data indicate that N6-methyladenosine (m^6^A) RNA methylation and lncRNA deregulation act crucial roles in cancer progression. Heterogeneous nuclear ribonucleoprotein A2B1 (HNRNPA2B1) as an m^6^A “reader” has been reported to be an oncogene in multiple malignancies. We herein aimed to elucidate the role and underlying mechanism by which HNRNPA2B1-mediated m^6^A modification of lncRNAs contributes to non-small cell lung cancer (NSCLC).

**Methods:**

The expression levels of HNRNPA2B1 and their association with the clinicopathological characteristics and prognosis in NSCLC were determined by RT-qPCR, Western blot, immunohistochemistry and TCGA dataset. Then, the role of HNRNPA2B1 in NSCLC cells was assessed by in vitro functional experiments and in vivo tumorigenesis and lung metastasis models. HNRNPA2B1-mediated m^6^A modification of lncRNAs was screened by m^6^A-lncRNA epi-transcriptomic microarray and verified by methylated RNA immunoprecipitation (Me-RIP). The lncRNA MEG3-specific binding with miR-21-5p was evaluated by luciferase gene report and RIP assays. The effects of HNRNPA2B1 and (or) lncRNA MEG3 on miR-21-5p/PTEN/PI3K/AKT signaling were examined by RT-qPCR and Western blot analyses.

**Results:**

We found that upregulation of HNRNPA2B1 was associated with distant metastasis and poor survival, representing an independent prognostic factor in patients with NSCLC. Knockdown of HNRNPA2B1 impaired cell proliferation and metastasis in vitro and in vivo, whereas ectopic expression of HNRNPA2B1 possessed the opposite effects. Mechanical investigations revealed that lncRNA MEG3 was an m^6^A target of HNRNPA2B1 and inhibition of HNRNPA2B1 decreased MEG3 m^6^A levels but increased its mRNA levels. Furthermore, lncRNA MEG3 could act as a sponge of miR-21-5p to upregulate PTEN and inactivate PI3K/AKT signaling, leading to the suppression of cell proliferation and invasion. Low expression of lncRNA MEG3 or elevated expression of miR-21-5p indicated poor survival in patients with NSCLC.

**Conclusions:**

Our findings uncover that HNRNPA2B1-mediated m^6^A modification of lncRNA MEG3 promotes tumorigenesis and metastasis of NSCLC cells by regulating miR-21-5p/PTEN axis and may provide a therapeutic target for NSCLC.

**Supplementary Information:**

The online version contains supplementary material available at 10.1186/s12967-023-04190-8.

## Introduction

Lung cancer is one of the most prevalent malignant tumors with the highest incidence and second cancer-related mortality worldwide [1]. Although great strides have been made to improve the cancer care, non-small cell lung cancer (NSCLC) as a subtype of lung cancer is provided with poor outcomes for advanced patients attributed to the tumor invasiveness and metastasis [2]. Dysregulated epigenetic modifications and noncoding RNAs are involved in cancer progression [3]. Therefore, comprehensive understanding of molecular mechanism underlying NSCLC metastasis is responsible for the effective therapeutic strategies.

N6-methyladenosine (m^6^A) has been considered as one of the most common chemical modifications in eukaryotic mRNAs and acts an essential role in cancer [4]. It has been verified that m^6^A methyltransferases such as METTL3/14 [5, 6], demethylases (FTO/ALKBH5) [7, 8] and m^6^A readers such as YTHDF1/2 and YTHDC2 [9, 10] participate in NSCLC carcinogenesis. HNRNPA2B1 as a nuclear reader is upregulated in esophageal cancer (ESCA) [11, 12], ovarian cancer [13], colorectal cancer (CRC) [14], multiple myeloma [15], oral squamous cell carcinoma (OSCC) [16], NSCLC [17] and associated with lymphatic metastasis and poor prognosis, acting as an oncogene in multiple malignancies [11–16]. The m^6^A-circRNA epi-transcriptomic microarray and MeRIP uncovered that METTL14 can mediate m^6^A-dependent modification of circORC5 in GC cells [6] and HNRNPA2B1 increases AKT3 expression by m^6^A-dependent stabilization of ILF3 mRNA [15]. However, HNRNPA2B1-mediated m^6^A modification in NSCLC is unclear.

It has been elucidated that lncRNA MEG3 is downregulated in prostate cancer [18], gastric cancer (GC) [19], pancreatic cancer [20], gallbladder cancer (GBC) [21], breast cancer [22], cervical cancer (CECA) [23], hepatocellular carcinoma (HCC) [24] and NSCLC [25], represses cell proliferation and invasion [18–25], and its low expression is associated with distant metastasis and poor prognosis [20–23, 25]. RIP assay showed that METTL3 mediates the m^6^A modification of MEG3 in HCC cells [26]. However, the role and mechanism underlying HNRNPA2B1-mediated m^6^A of lncRNA MEG3 in NSCLC remain undocumented.

The miRNA-seq or m^6^A-related lncRNA analysis revealed that HNRNPA2B1 can act by regulating miRNAs and lncRNAs in cancer [27, 28]. In the present study we found that HNRNPA2B1 upregulation was associated with distant metastasis, acting as an independent prognostic factor for poor survival in patients with NSCLC and HNRNPA2B1-mediated m^6^A modification of lncRNA MEG3 promoted tumorigenesis and metastasis of NSCLC cells by regulating miR-21-5p/PTEN axis, providing a promising therapeutic target for NSCLC.

## Materials and methods

### Clinical samples

The clinical data for 455 cases of lung adenocarcinoma (LAC) patients and 59 pair-matched tumor tissue samples were downloaded from The Cancer Genome Atlas database (http://xena.ucsc.edu/getting-started/). The data for 407 cases of LAC patients included clinicopathological and prognostic information such as age, sex, TNM stage, pathological stage, survival time and survival status. 10 pairs of LAC tissue samples were stored in liquid nitrogen and frozen at −80 °C. A tissue microarray containing 80 pair-matched LAC tissues (Lot No. LUC1601) was purchased from Shanghai Outdo Biotech Company (Shanghai, China). Our study protocol was approved by the Ethics Committee of Yunnan Cancer Hospital.

### Bioinformatic analysis

The association of HNRNPA2B1, lncRNA MEG3 and miR-21-5p with the prognosis of lung cancer patients could be analyzed using the GEO dataset by Kaplan–Meier Plotter (http://kmplot.com/analysis/index.php?p=service&cancer=lung). The miRNA-lncRNA interaction and miRNA-target interaction could be analyzed by starBaseV2.0 (https://starbase.sysu.edu.cn/starbase2/mirLncRNA.php).

### Immunohistochemical (IHC) analysis

The tissue microarray (Lot No. LUC1601) was fixed with 4% paraformaldehyde and was trimmed, dehydrated, embedded, sliced, stained and sealed in strict accordance with the SOP procedure for pathological test of the unit. The slides were incubated with mouse anti-HNRNPA2B1 monoclonal antibody (Proteintech, 1:200, Lot No. 67445-1-Ig, Wuhan, China) and rabbit anti-Ki-67 (ab833, abcam), and the protein expression of HNRNPA2B1 was examined by two independent pathologists according to the H-Score. H-Score = ∑ (PI × I) = (percentage of cells of weak intensity × 1) + (percentage of cells of moderate intensity × 2) + (percentage of cells of strong intensity × 3).

### RNA extraction and real-time quantitative PCR (RT-qPCR)

Total RNA was extracted using Trizol (Ambion, 15596-026) and cDNA synthesis was performed using HiScript^®^ II Q Select RT SuperMix (VAZYME, R233), SYBR Green Master Mix (VAZYME, Q111-02,) and gene primers (Additional file 5: Table S6). After the PCR reactions were finished, the relative expression levels of HNRNPA2B1, lncRNA MEG3, miR-21-5p and PTEN were quantified using the 2^−ΔΔCt^.

### Western blot analysis

LAC tissue and cell lines were lysed with RIPA buffer. The supernatants were resolved in SDS-PAGE and transferred onto polyvinylidene fluoride membranes (IPVH00010, Millipore, MA, USA), which were then probed with anti-HNRNPA2B1 (Proteintech, 1:200, Lot No. 67445-1-Ig, Wuhan, China), anti-PTEN (Proteintech, 1:1000, 60300-1-Ig, Wuhan, China), anti-PI3K (1:1000, AF6241, Affinity), anti-p-PI3K (1:1000, AF3241, Affinity), anti-AKT (Proteintech, 1:1000, 10176-2-AP, Wuhan, China), anti-p-AKT (Proteintech, 1:1000, 66444-1-Ig, Wuhan, China), anti-PCNA (1:1000, GB11010, Servicebio), anti-MMP2 (1:1000, AF5330, Affinity) and anti-GAPDH (1:1000, AB-P-R 001, GOODHERE, Hangzhou, China) overnight at 4 °C. Protein bands were emerged by enhanced chemiluminescence method.

### Plasmid, shRNA, miRNA mimic and inhibitor

HNRNPA2B1 overexpression plasmids, lentivirus-mediated HNRNPA2B1 shRNA (sh-HNRNPA2B1, 5ʹ-CCGTAAGCTCTTTATTGGTGGCTTA-3ʹ), si-MEG3, miR-21-5p mimics and inhibitor were purchased from GenePharma (Shanghai, China). The sh-NC, vector and miR-NC were used as the control groups. NSCLC cell lines were planted in 6-well plates 24 h prior to sh-HNRNPA2B1, si-MEG3, miR-21-5p mimics or inhibitor transfection with 50–60% confluence, and then mixed with Lipofectamine 2000 (Invitrogen, Carlsbad, CA, USA) according to the manufacture instructions.

### Cell culture, MTT, colony formation, transwell assays

These assays were conducted as previously reported [6, 17, 28].

### Human M^6^A-lncrna epi-transcriptomic microarray

After 95D cell liens were transfected with sh-HNRNPA2B1 or sh-NC lentiviruses for 48 h, the total RNA from each sample was quantified using the NanoDrop ND-1000. Briefly, the total RNAs were immunoprecipitated with anti- m^6^A antibody. The modified RNAs were eluted from the immunoprecipitated magnetic beads as the “IP”. The unmodified RNAs were recovered from the supernatant as “Sup”. The “IP” and “Sup” RNAs were treated with RNase R, and then labeled with Cy5 and Cy3 respectively as cRNAs in separate reactions using Arraystar Super RNA Labeling Kit. The cRNAs were combined together and hybridized onto Arraystar Human lncRNA Epi-transcriptomic Microarray. After washing the slides, the arrays were scanned in two-color channels by an Agilent Scanner G2505C.

### RNA immunoprecipitation (RIP) and m.^6^A RIP (MeRIP)

RIP assay was carried out in 95D and H1299 cell lines using a Magna RIP RNA-binding protein Immunoprecipitation Kit (Millipore) according to the manufacturer’s instructions. Antibodies against Ago2 and IgG for RIP assays were purchased from Abcam (ab5072, Cambridge, MA, USA). Anti-m^6^A antibody (A-1801–020, Epigentek) were used for MeRIP assay.

### Luciferase reporter assay

95D and H1299 cells were seeded into 96-well plates and co-transfected with PRL-TK-pMIR-MEG3 or PRL-TK-pMIR-PTEN 3’UTR, and miR-21-5p mimics or miR-NC. After 48 h of incubation, the firefly and Renilla luciferase activities were examined with a dual-luciferase reporter assay.

### In vivo tumorigenesis model

Male nude mice (6 weeks old) were purchased from the Shanghai Laboratory Animal Central (Shanghai, China). 95D cells (1 × 10^7^) transfected with sh-HNRNPA2B1 or sh-NC lentiviruses were injected subcutaneously into the right flanks of mice. After 8 weeks, the mice were sacrificed, and the xenografted tumors were collected for hematoxylin–eosin (HE) staining and IHC analysis. The animal experiments were approved by the Ethics Committee of Yunnan Cancer Hospital.

### Caudal vein pulmonary metastasis model

95D cells stably transfected with sh-HNRNPA2B1 or sh-NC lentiviruses were cultured in complete medium. When the cells were 70% confluent, the medium was replaced with fresh medium to remove dead and detached cells. 1 × 10^7^ 95D cells were injected into the mice tail vein. The progression of pulmonary metastasis was investigated for 5 weeks.

### Hematoxylin and eosin (HE) staining

Mice tumor tissues were harvested and fixed in 4% paraformaldehyde, and preserved in optimal cutting temperature compound. The lung tissues were sliced in 5 μm sections and stained with HE for the histological studies.

### Statistical analysis

Statistical analyses were conducted with GraphPad Prism 7 (La Jolla, CA, USA). The values are indicated as the mean ± standard deviation. Student’s t test and analysis of variance were used for comparisons between groups. Kaplan–Meier analysis was used to assess the association of HNRNPA2B1, lncRNA MEG3 and miR-21-5p with the prognosis in patients with NSCLC. A Cox proportional hazard model was used to assess the risk of HNRNPA2B1 in NSCLC. *P* < 0.05 was considered statistically significant.

## Results

### Elevated expression of HNRNPA2B1 was associated with poor survival in patients with NSCLC

**Fig. 1:**
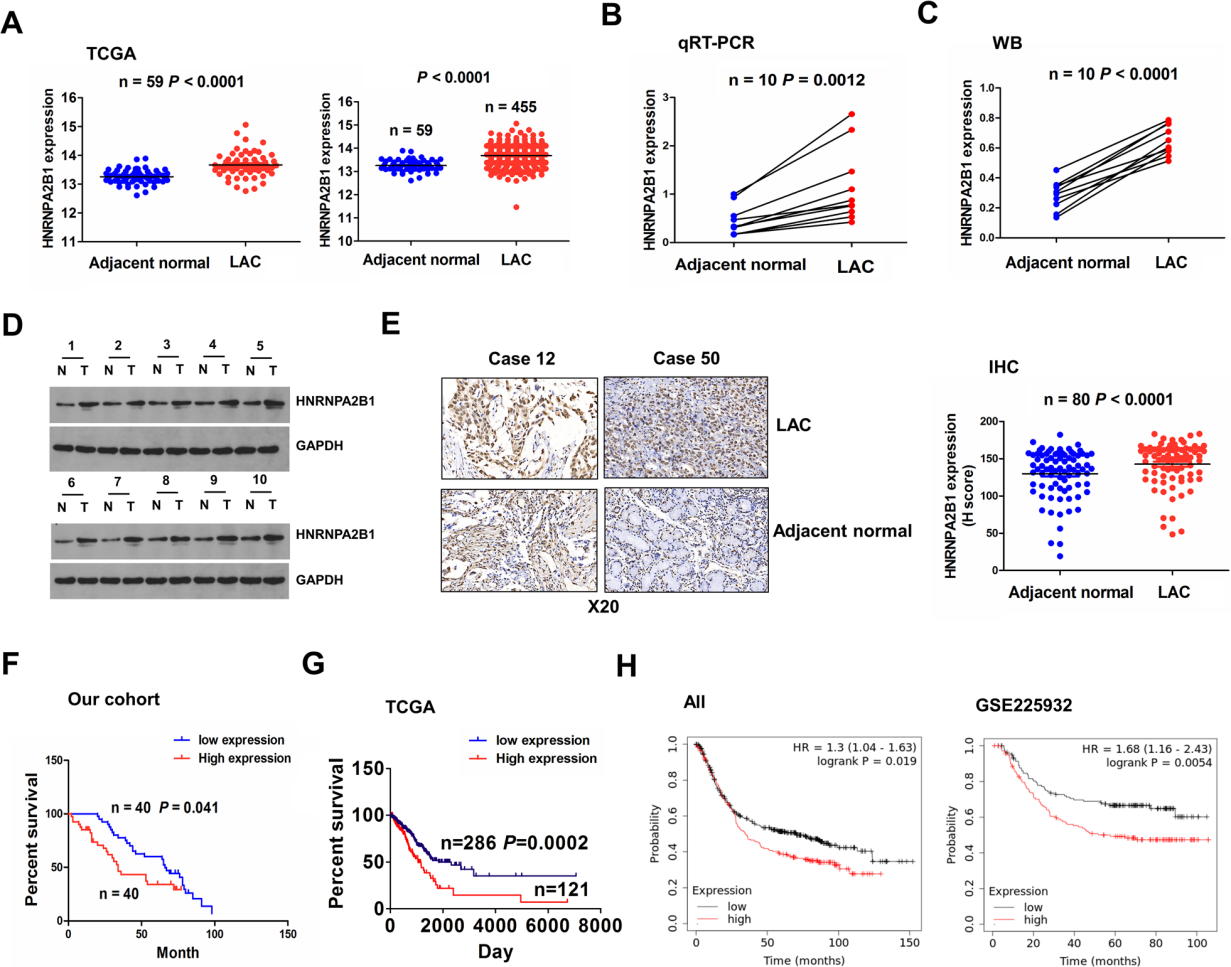
Upregulation of HNRNPA2B1 was associated with poor survival in patients with NSCLC. **A** TCGA cohort analysis of the expression levels of HNRNPA2B1 in pair-matched and non-paired NSCLC tissues. **B** RT-qPCR and **C**, **D** Western blot analysis of the expression levels of HNRNPA2B1 in 10 pairs of NSCLC tissue samples. **E** IHC analysis of the protein expression of HNRNPA2B1 in 80 pairs of NSCLC tissue samples. **F** Our cohort, **G** TCGA cohort and **H** GEO cohort analysis of the association of HNRNPA2B1 expression with the prognosis of NSCLC patients

To unveil the role of HNRNPA2B1 in NSCLC, we utilized the TCGA cohort to investigate the expression of HNRNPA2B1 in NSCLC and found that HNRNPA2B1 was dramatically upregulated in pair-matched (n = 59) and non-paired LAC tissue samples (n = 455) as compared with the adjacent normal tissues (n = 59, Fig. 1A). The similar results for HNRNPA2B1 expression were validated in 10 pairs of LAC tissue samples by RT-qPCR and Western blot analyses (Fig. 1B–D). IHC analysis further indicated that HNRNPA2B1 expression levels were markedly increased in 80 pairs of NSCLC tissues samples relative to the adjacent normal tissues (Fig. 1E). In addition, we found that elevated expression of HNRNPA2B1 was associated with age and distant metastasis in patients with NSCLC (Additional file 5: Table S1). Kaplan–Meier analysis from our cohort implied that the patients with high-HNRNPA2B1 expression harbored poorer survival rate as compared with those with low-HNRNPA2B1 expression (Fig. 1F). Another two independent cohorts from the TCGA and GEO cohorts indicated that the patients with high-HNRNPA2B1 group displayed worse survival in patients with NSCLC (Fig. 1G, H). Multivariate Cox regression analysis uncovered that high expression of HNRNPA2B1 as well as lymph node infiltration constituted an independent prognostic factor for poor survival in patients with NSCLC (Additional file 5: Table S2). These results indicated that HNRNPA2B1 was a trustworthy prognostic factor for NSCLC patients.

### Knockdown of HNRNPA2B1 repressed the proliferation and invasion of NSCLC cells

**Fig. 2:**
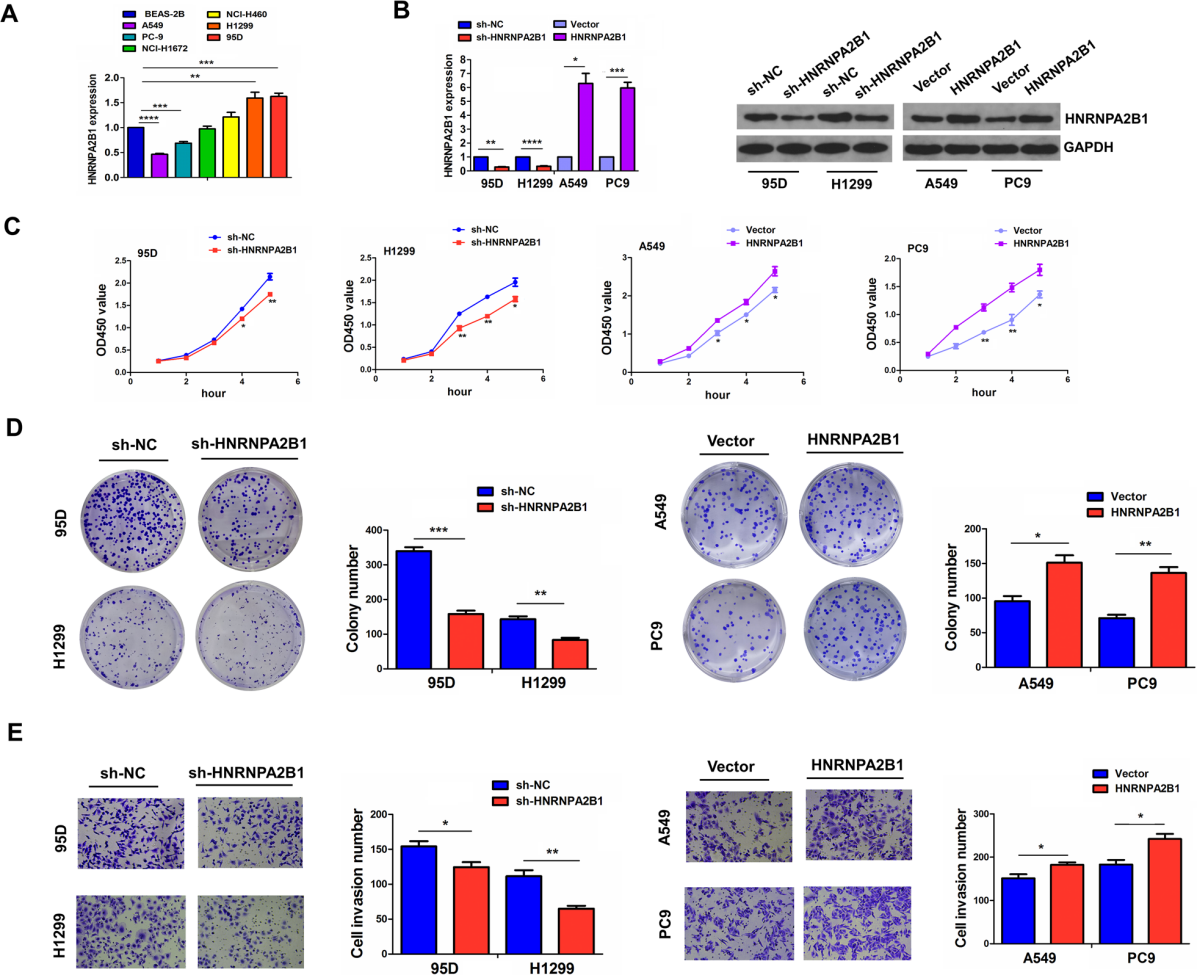
Knockdown of HNRNPA2B1 repressed growth and invasion of NSCLC cells. **A** RT-qPCR analysis of the mRNA expression levels of HNRNPA2B1 in BEAS-2B and NSCLC cell lines. **B** RT-qPCR and Western blot analysis of the transfection efficiency of sh-HNRNPA2B1 in 95D and H1299 cells or HNRNPA2B1 in A549 and PC9 cells. **C** MTT analysis of the effects of HNRNPA2B1 knockdown or overexpression on the cell viability of NSCLC cells. **D** Colony formation analysis of the effects of HNRNPA2B1 knockdown or overexpression on the colony formation ability of NSCLC cells. **E** Transwell analysis of the effects of HNRNPA2B1 knockdown or overexpression on the cell invasion of NSCLC cells. Data are the means ± SEM of three experiments. *P < 0.05; **P < 0.01; ***P < 0.001; ****P < 0.0001

Having confirmed that HNRNPA2B1 was upregulated in NSCLC, we hypothesized that HNRNPA2B1 might act as an oncogene in NSCLC. We then examined the mRNA levels of HNRNPA2B1 in BEAS-2B and NSCLC cell lines by RT-qPCR and found that HNRNPA2B1 showed increased expression in 95D and H1299 cell lines, but decreased expression in A549 and PC-9 cell lines (Fig. 2A). To elucidate the role of HNRNPA2B1 in NSCLC, we constituted HNRNPA2B1-kockdown cell models in 95D and H1299 cell lines with shRNA, and HNRNPA2B1-overexpression cell models in A549 and PC-9 cell lines with plasmids. The transfection efficiency was verified by RT-qPCR and Western blot (Fig. 2B). Further functional assays indicated that knockdown of HNRNPA2B1 inhibited the cell viability (Fig. 2C), cell colony formation (Fig. 2D) and cell invasion abilities (Fig. 2E) in 95D and H1299 cell lines, whereas ectopic expression of HNRNPA2B1 promoted these effects in A549 and PC-9 cell lines (Fig. 2C–E).

### HNRNPA2B1 acted by modification of lncRNA MEG3 in an m^6^A-dependent manner

**Fig. 3:**
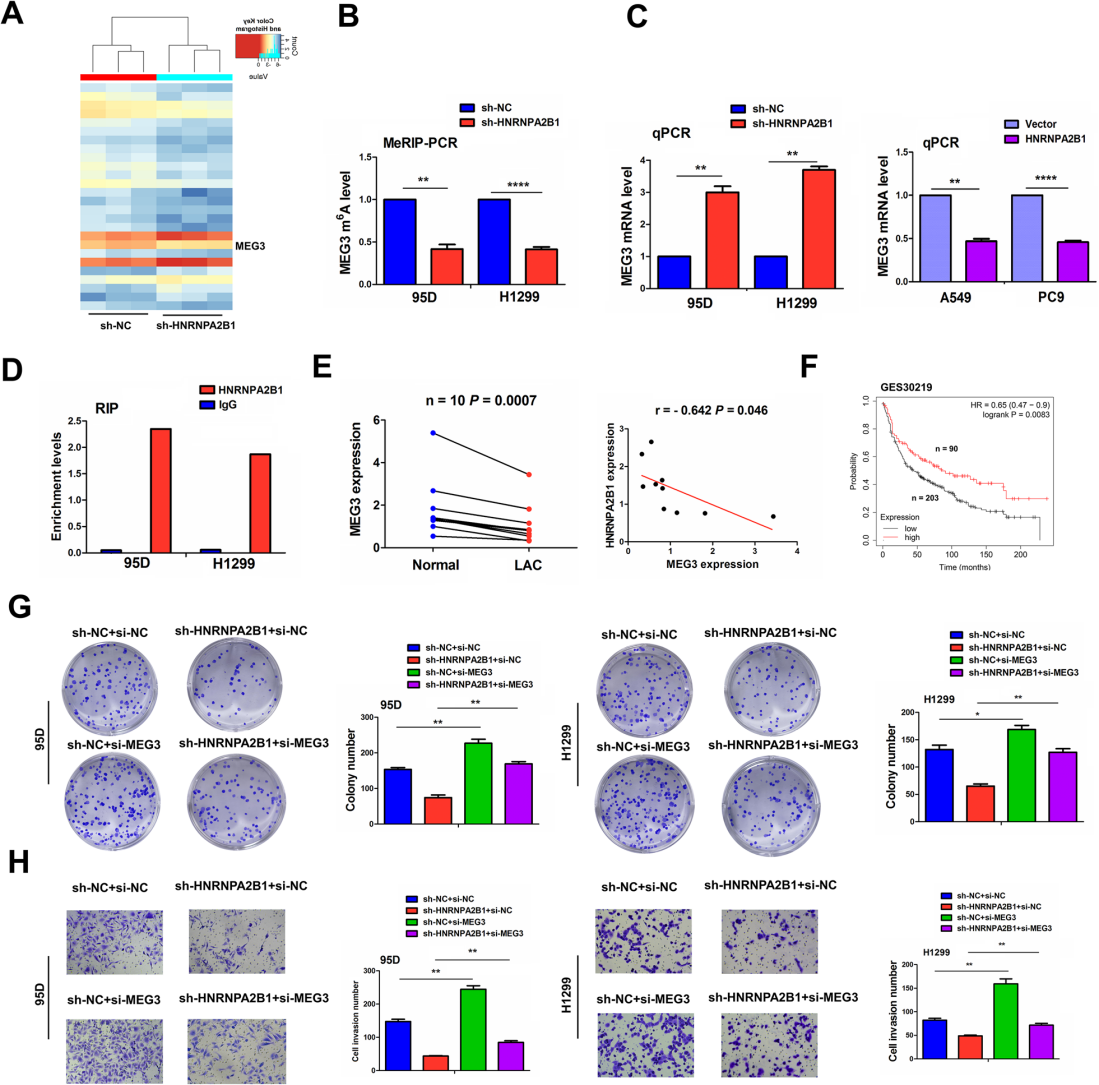
HNRNPA2B1 acted by m^6^A-dependent modification of lncRNA MEG3 in NSCLC cells. **A** m^6^A-lncRNA microarray identification of m^6^A-modified lncRNAs between sh-HNRNPA2B1 and sh-NC transfected 95D cells. **B** MeRIP analysis of the effects of HNRNPA2B1 knockdown on the m.^6^A levels of lncRNA MEG3 in 95D and H1299 cells. **C** RT-qPCR analysis of the effects of HNRNPA2B1 knockdown or overexpression on the expression levels of lncRNA MEG3 in 95D and H1299 cells. **D** RIP analysis of the binding between HNRNPA2B1 and lncRNA MEG3 in 95D and H1299 cells. **E** RT-qPCR analysis of the expression levels of lncRNA MEG3 and its correlation with HNRNPA2B1 in 10 pairs of NSCLC tissues. **F** GES30219 analysis of the association of lncRNA MEG3 expression with the prognosis of NSCLC patients. **G** Colony formation and **H** Transwell assays were used to estimate the effects of co-transfection with sh-HNRNPA2B1 and (or) si-MEG3 on cell proliferation and invasion in 95D and H1299 cells. Data are the means ± SEM of three experiments. *P < 0.05; **P < 0.01. ****P < 0.0001

To underline the molecular mechanism of HNRNPA2B1 in NSCLC, we used m^6^A-lncRNA epi-transcriptomic microarray to identify HNRNPA2B1-mediated m^6^A modification of lncRNAs between sh-HNRNPA2B1 and sh-NC transfected 95D cells and found that the m^6^A levels of 20 lncRNAs were deceased but those of 6 lncRNAs were increased in HNRNPA2B1-knockdown 95D cells (Fig. 3A). Among these lncRNAs, we found that lncRNA MEG3 had the most obvious downregulation in m^6^A levels in HNRNPA2B1-knockdown 95D cells. Furthermore, MeRIP-PCR validated that the m^6^A levels of lncRNA MEG3 were remarkably lowered by knockdown of HNRNPA2B1 in 95D and H1299 cell lines (Fig. 3B). RT-qPCR analysis indicated that lncRNA MEG3 expression was significantly elevated by HNRNPA2B1 knockdown in 95D and H1299 cell lines but reduced by HNRNPA2B1 overexpression in A549 and PC-9 cell lines (Fig. 3C). We further carried out RIP assay for HNRNPA2B1 in 95D and H1299 cells and found that the endogenous levels of lncRNA MEG3 pulled down from HNRNPA2B1 protein were mainly enriched in the HNRNPA2B1 pellet compared to those in the input control (Fig. 3D). The expression of lncRNA MEG3 indicated by RT-qPCR analysis was diminished and had a negative correlation with HNRNPA2B1 expression in 10 pairs of LAC tissues (Fig. 3E). Low expression of lncRNA MEG3 was associated with pathological stage and lymph node infiltration in patients with NSCLC (Additional file 5: Table S3). Kaplan–Meier analysis from GES30219, GES14814 and GES37745 cohorts suggested that the patients with high-MEG3 expression had more favorable survival compared to those with low-MEG3 expression (Fig. 3F and Additional file 2: Figure S2).

The transfection efficiency of si-MEG3 in 95D and H1299 cells was acknowledged by RT-qPCR analysis (Additional file 1: Figure S1). Functional assays indicated that knockdown of MEG3 facilitated cell colony formation and cell invasion abilities and reversed HNRNPA2B1 knockdown-induced antitumor effects in 95D and H1299 cells (Fig. 3G, H). These results implied that HNRNPA2B1 acted by modification of lncRNA MEG3 in an m^6^A-dependent manner.

### LncRNA MEG3 acted as a sponge of miR-21-5p in NSCLC cells

**Fig. 4:**
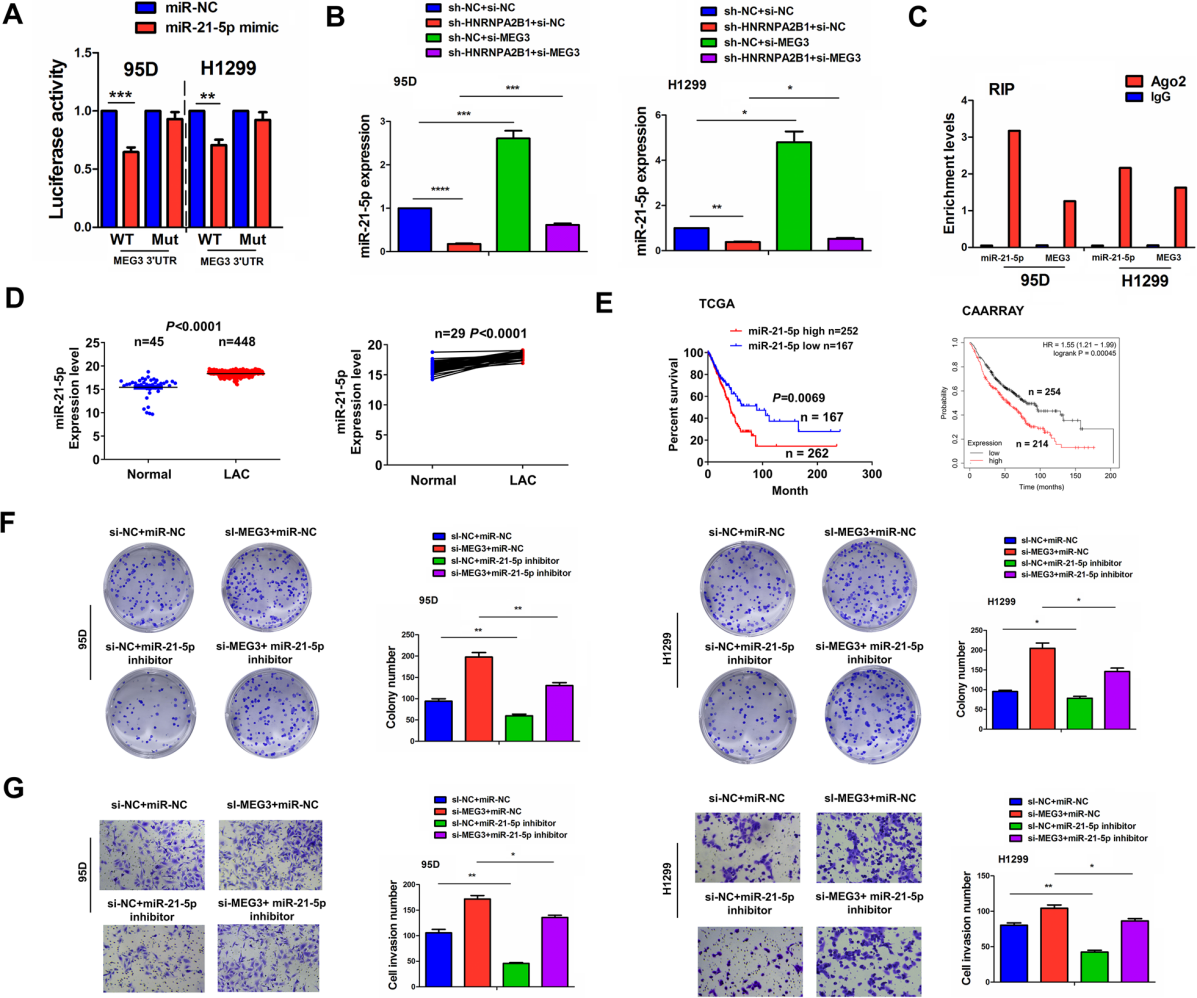
LncRNA MEG3 acted as a sponge of miR-21-5p in NSCLC cells. **A** The luciferase activity of the WT luc-MEG3 or Mut luc-MEG3 after transfection with miR-21-5p mimics in 95D and H1299 cells. **B** RT-qPCR analysis of the effects of co-transfection with sh-HNRNPA2B1 and (or) si-MEG3 on miR-21-5p expression in 95D and H1299 cells. **C** RIP for Ago2 protein was used to assess the endogenous expression of lncRNA MEG3 and miR-21-5p in 95D and H1299 cells. **D** TCGA cohort analysis of the expression levels of miR-21-5p in pair-matched and non-paired NSCLC tissues. **E** TCGA and CAARRAY analysis of the association of miR-21-5p expression with the prognosis of NSCLC patients. **F** Colony formation and **G** Transwell analysis of the effects of co-transfection with miR-21-5p inhibitor and (or) si-MEG3 on cell proliferation and invasion in 95D and H1299 cells. Data are the means ± SEM of three experiments. *P < 0.05; **P < 0.01, ***P < 0.001, ****P < 0.0001

Emerging data have shown that lncRNA MEG3 can act as a tumor suppressor by sponging miR-21-5p [29, 30]. The binding sites between lncRNA MEG3 and miR-21-5p can be indicated in Additional file 3: Figure S3. The transfection efficiency of miR-21-5p mimics or inhibitor in 95D and H1299 cells was determined by RT-qPCR (Additional file 4: Figure S4). We then found that miR-21-5p mimics could lower the luciferase activity of WT MEG3 3’ UTR, but displayed no impact on that of Mut MEG3 3’-UTR compared to miR-NC group in 95D and H1299 cells (Fig. 4A). RT-qPCR analysis showed that knockdown of lncRNA MEG3 increased the expression of miR-21-5p and reversed HNRNPA2B1-knockdown induced miR-21-5p downregulation in 95D and H1299 cells (Fig. 4B). RIP assay for Ago2 further validated that the endogenous levels of lncRNA MEG3 and miR-21-5p pulled down from Ago2 protein were enriched in the Ago2 pellet compared to those in the input control (Fig. 4C). TCGA cohort showed that miR-21-5p was upregulated in pair-matched and non-paired NSCLC tissues as compared with the adjacent normal tissues (Fig. 4D). Elevated expression of miR-21-5p was associated with lymph node infiltration in patients with NSCLC (Additional file 5: Table S4). Kaplan–Meier analysis from TCGA and CAARRAY cohorts revealed that the patients with high-miR-21-5p expression possessed poorer survival compared to those with low-miR-21-5p expression (Fig. 4E). However, miR-21-5p expression was not an independent prognostic factor for poor survival in patients with NSCLC (Additional file 5: Table S5). Functional assays demonstrated that miR-21-5p inhibitor suppressed cell colony formation and invasion abilities and counteracted MEG3-knockdown induced tumor-promoting effects in 95D and H1299 cells (Fig. 4F, G).

### HNRNPA2B1 mediated lncRNA MEG3 to regulate miR-21-5p/PTEN axis

**Fig. 5:**
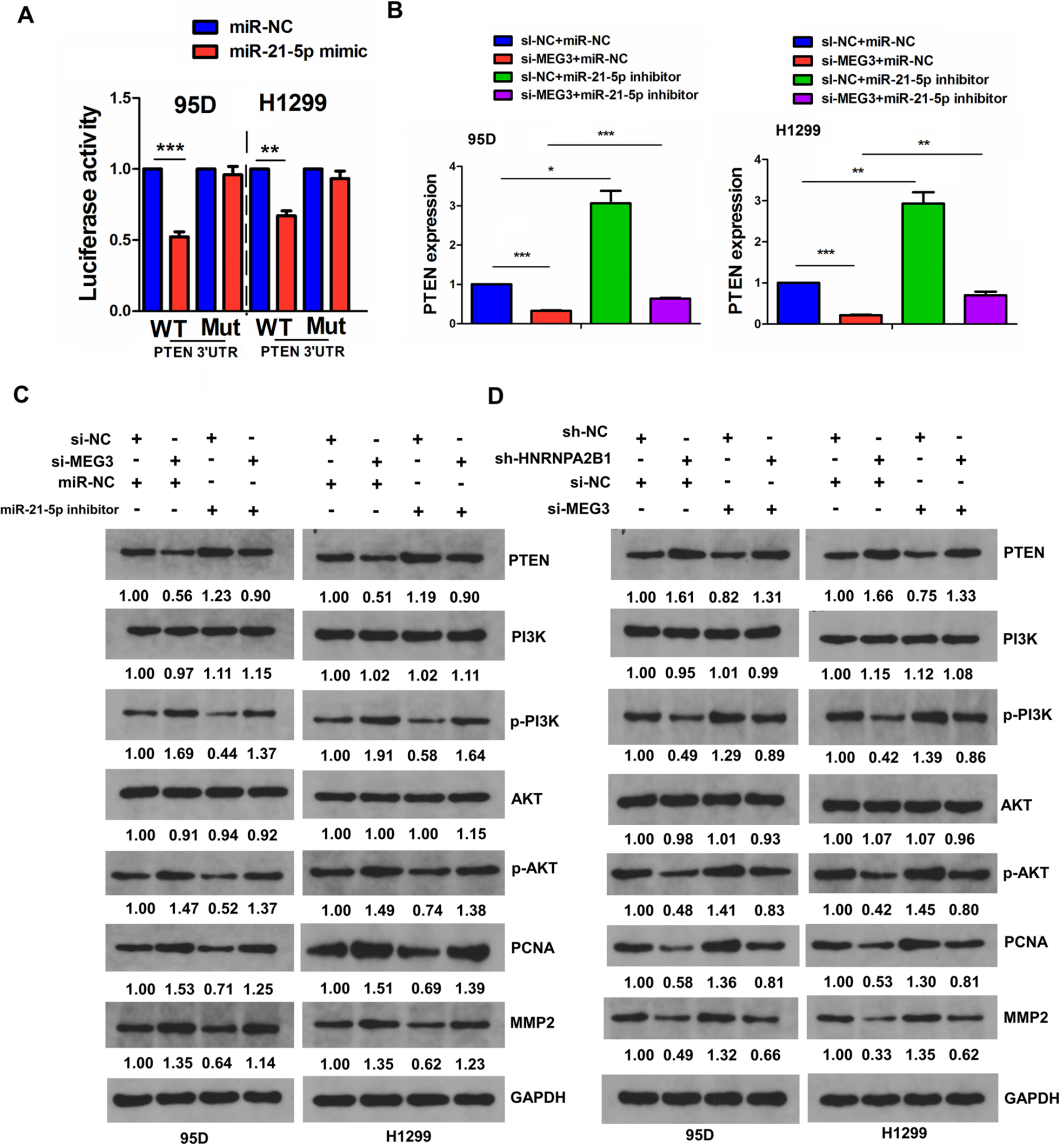
HNRNPA2B1 mediated lncRNA MEG3 to regulate miR-21-5p/PTEN axis in NSCLC cells. **A** The luciferase activity of the WT luc-PTEN or Mut luc-PTEN after transfection with miR-21-5p mimics in 95D and H1299 cells. **B** RT-qPCR analysis of the effects of co-transfection with miR-21-5p inhibitor and (or) si-MEG3 on PTEN expression in 95D and H1299 cells. **C** Western blot analysis of the effects of co-transfection with miR-21-5p inhibitor and (or) si-MEG3 on PTEN/PI3K/AKT signaling activity in 95D and H1299 cells. **D** Western blot analysis of the effects of co-transfection with sh-HNRNPA2B1 and (or) si-MEG3 on PTEN/PI3K/AKT signaling activity in 95D and H1299 cells

Increasing evidence manifests that miR-21-5p/PTEN axis is involved in carcinogenesis [31–33]. We found that miR-21-5p mimics could inhibit the luciferase activity of WT PTEN 3’ UTR, but exhibited no effect on that of the Mut PTEN 3’UTR compared to the miR-NC group in 95D and H1299 cells (Fig. 5A). Moreover, RT-qPCR and Western blot analyses showed that miR-21-5p inhibitor increased the expression of PTEN and attenuated MEG3-knockdown induced PTEN downregulation in 95D and H1299 cells (Fig. 5B, C). Also, miR-21-5p inhibitor inhibited the activation of PI3K/AKT signaling and counteracted MEG3-knockdown induced PI3K/AKT signaling activation in 95D and H1299 cells (Fig. 5C). In contrast, knockdown of MEG3 deceased the expression of PTEN but promoted PI3K/AKT signaling activation and abolished HNRNPA2B1-knockdown induced PTEN upregulation and PI3K/AKT signaling inactivation in 95D and H1299 cells (Fig. 5D).

### Knockdown of HNRNPA2B1 repressed in vivo tumorigenesis

**Fig. 6:**
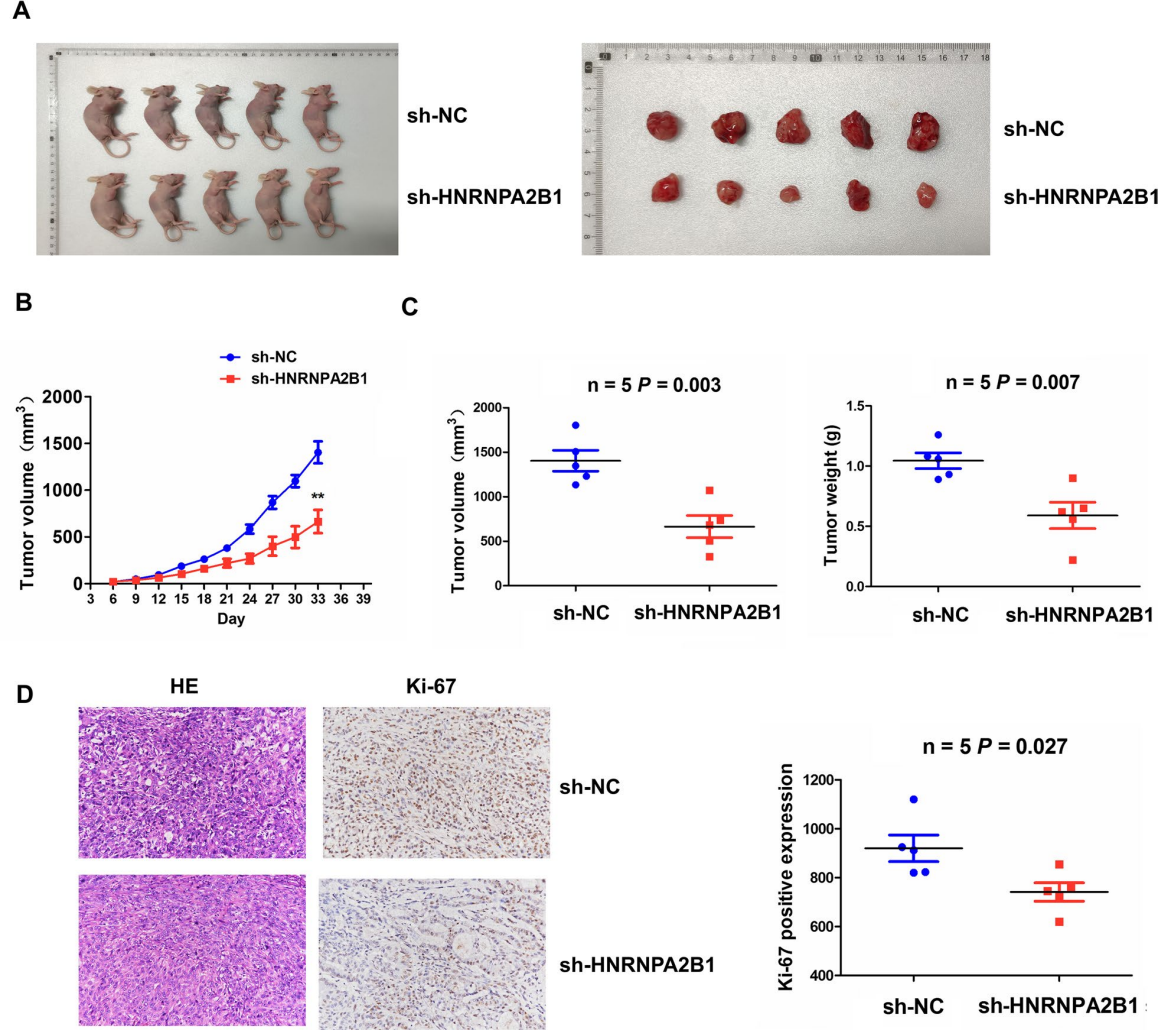
Knockdown of HNRNPA2B1 suppressed in vivo NSCLC tumorigenesis. **A** Schematic representation of the xenograft tumors between sh-HNRNPA2B1 and sh-NC transfected groups. **B** A tumor growth curve analysis of the tumor growth trend in sh-HNRNPA2B1 and sh-NC transfected groups. **C** Comparison of the tumor volume and weight between sh-HNRNPA2B1 and sh-NC transfected groups. **D** H&E and IHC analysis of Ki-67 expression in sh-HNRNPA2B1 and sh-NC transfected groups. Data are the means ± SEM of five experiments

To clarify whether HNRNPA2B1 influences in vivo NSCLC tumorigenesis, we utilized sh-HNRNPA2B1 or sh-NC stably transfected 95D cells to construct the xenograft tumor models by subcutaneous injection into the flank of nude mice. When the mice were sacrificed, we found that the volumes of xenograft tumors formed by sh-HNRNPA2B1 transfected 95D cells were smaller than those by sh-NC-transfected cells (Fig. 6A). Tumor growth curve showed that the xenograft tumors in sh-HNRNPA2B1 group presented a relatively slow growth trend in a time-dependent manner (Fig. 6B), and both of the tumor volume and weight were dwindled in sh-HNRNPA2B1 group as compared with the sh-NC group (Fig. 6C). HE and IHC analyses demonstrated that the expression levels of Ki-67, a proliferation-related tumor marker were reduced in sh-HNRNPA2B1 group as compared with the NC group (Fig. 6D).

### Knockdown of HNRNPA2B1 suppressed in vivo lung metastasis

**Fig. 7:**
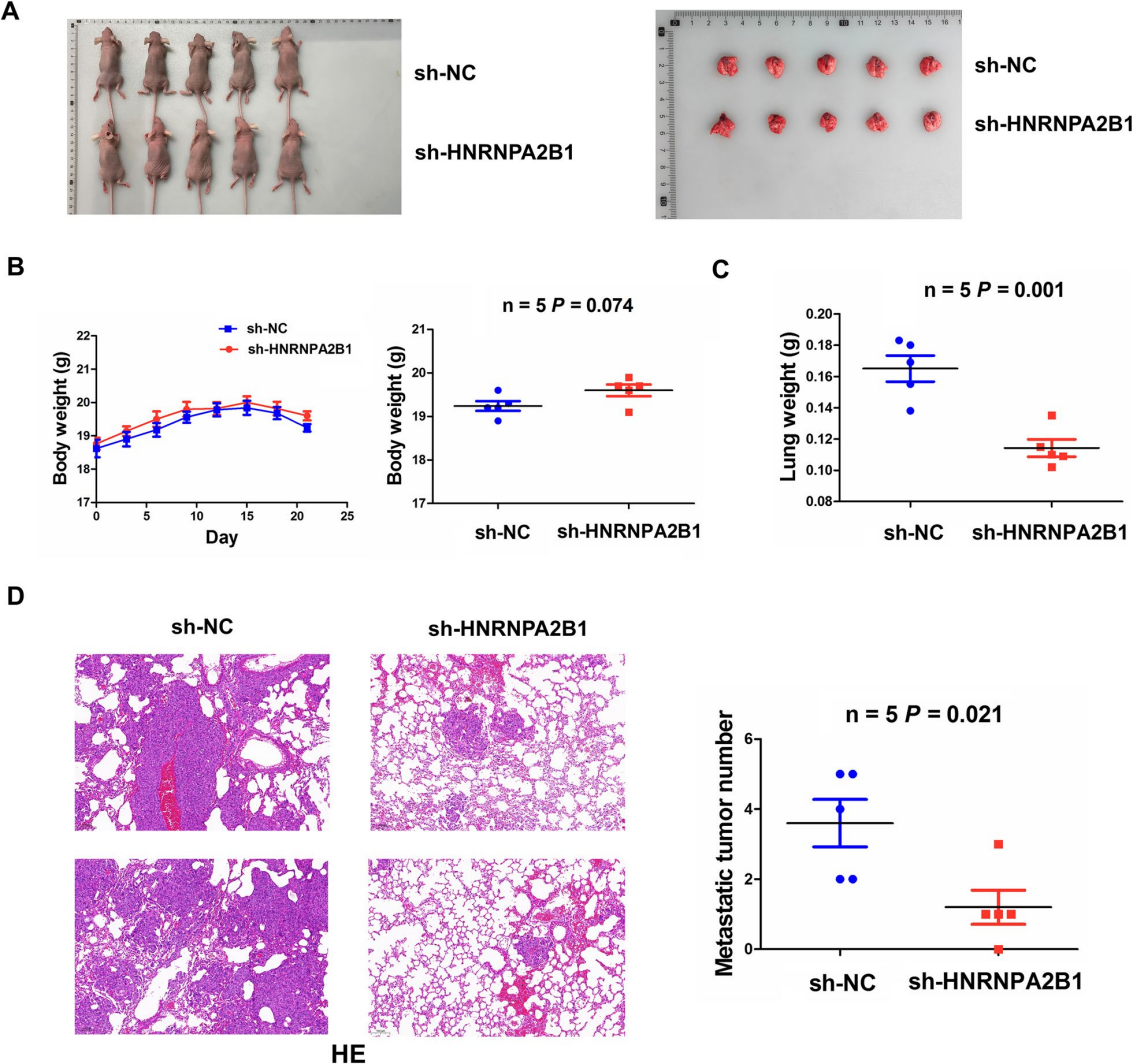
Knockdown of HNRNPA2B1 inhibited in vivo NSCLC metastasis. **A** Schematic representation of the lung metastasis models between sh-HNRNPA2B1 and sh-NC transfected groups. **B** A body weight curve analysis of the body weight trend in sh-HNRNPA2B1 and sh-NC transfected groups. **C** Comparison of the lung weight between sh-HNRNPA2B1 and sh-NC transfected groups. **D** H&E analysis of metastatic tumor number in sh-HNRNPA2B1 and sh-NC transfected groups. Data are the means ± SEM of five experiments

To pinpoint whether HNRNPA2B1 affects in vivo NSCLC metastasis, we utilized sh-HNRNPA2B1 or sh-NC stably transfected 95D cells to establish the lung metastasis models through injection of the tail vein of mice (Fig. 7A). When the mice were sacrificed, we found that the body weight of the mice had no significant difference between sh-HNRNPA2B1 and sh-NC groups (Fig. 7B), but the lung weight was dramatically declined in sh-HNRNPA2B1 group as compared with the sh-NC group (Fig. 7C). HE analysis demonstrated that the number of metastatic tumor nodules was decreased in sh-HNRNPA2B1 group as compared with the NC group (Fig. 7D).

## Discussion

Accumulating evidence unveils that HNRNPA2B1 as a m^6^A reader harbors an increased expression in multiple malignances including NSCLC [11–18] and indicates poor prognosis in patients with NSCLC [17, 34]. In accordance with these studies, we also discovered that HNRNPA2B1 was upregulated in NSCLC tissue samples and associated with distant metastasis and unfavorable overall survival in patients with NSCLC, implying that HNRNPA2B1 might be a prognostic factor for poor survival in NSCLC. Further investigations indicated that HNRNPA2B1 acted a pivotal role in promoting NSCLC proliferation and invasion. In addition, we found that lncRNA MEG3 was a downstream target of m^6^A modification mediated by HNRNPA2B1. Our findings suggested that HNRNPA2B1-mediated m^6^A modification promoted NSCLC progression by regulating lncRNA MEG3 expression at the post-transcriptional level. Our results might offer a new epigenetic regulatory mechanism leading to the progression of NSCLC.

Previous reports showed that HNRNPA2B1 acts a carcinogenic role in a variety of malignancies. HNRNPA2B1 can promote tumorigenesis and metastasis by multiple pathways such as regulating ACLY expression [11] and Lin28B stability [13], activating ERK/MAPK signaling [14], modulating miR-17–92/miR-9-5p/miR-21 [12, 18, 19] and mediating m^6^A-dependent stabilization of ILF3 [15]. Meanwhile, HNRNPA2B1 can interact with cyclooxygenase-2 [34] and miR-122-5p to enhance NSCLC progression [35]. In the present study, we found that knockdown of HNRNPA2B1 impaired proliferation and metastasis of NSCLC cells in vitro and in vivo, whereas overexpression of HNRNPA2B1 reversed these effects. The m^6^A-circRNA microarray and MeRIP have been shown to represent solid methods for screening m^6^A-modified mRNAs or noncoding RNAs in cancer [^6^, ^15^]. In our study, m^6^A-lncRNA microarray and MeRIP revealed that HNRNPA2B1 could display m^6^A-dependent modification of lncRNA MEG3 in NSCLC cells. Inhibition of HNRNPA2B1 could reduce the m^6^A level of lncRNA MEG3 but increased its expression. Knockdown of lncRNA MEG3 encouraged cell proliferation and invasion and counteracted HNRNPA2B1-knockdown induced antitumor effects. Our findings suggested that HNRNPA2B1 might act as an oncogene in NSCLC by m^6^A-dependent modification of lncRNA MEG3.

Increasing data support that lncRNA MEG3 acts as a tumor suppressor in cancers including NSCLC [18–25]. It can inhibit tumor progression by promoting EZH2 ubiquitination [21], activating PKM2 and inactivating PTEN [24] and sponging miR-421 [22]. Herein, we found that lncRNA MEG3 was downregulated in NSCLC samples and associated with favorable survival in patients with NSCLC. lncRNA MEG3 could be bound with miR-21-5p and knockdown of MEG3 incremented miR-21-5p expression and reversed HNRNPA2B1-knockdown induced miR-21-5p downregulation in NSCLC cells. It has been shown that miR-21-5p is upregulated in melanoma [36], NSCLC [37], GC [38] and promotes tumor proliferation and invasion by targeting CDKN2C/SET/TAF-Iα [34, 35]. We herein found that miR-21-5p expression was increased in NSCLC tissue samples and harbored a poor prognosis in patients with NSCLC. Inhibition of miR-21-5p restrained cell colony formation and invasion and abolished MEG3-knockdown caused tumor-promoting effects in NSCLC cells. Studies have shown that lncRNA HAND2-AS1, LINC00968 and MEG3 can suppress CECA and NSCLC progression by sponging miR-21-5p [39–41]. Our findings indicated that HNRNPA2B1-mediated m^6^A modification of lncRNA MEG3 impaired NSCLC progression by sponging miR-21-5p.

The inactivation of phosphatase and tensin homolog (PTEN) leads to epithelial-mesenchymal transition and metastasis of NSCLC [42] and PTEN/PI3K/AKT signaling is implicated in the regulation of NSCLC tumorigenesis [43]. We herein identified PTEN as a direct target of miR-21-5p, and miR-21-5p inhibitor upregulated PTEN and inactivated PI3K/AKT signaling and reversed MEG3-knockdown induced PTEN downregulation and PI3K/AKT signaling activation in NSCLC cells. Likewise, MEG3 knockdown abolished HNRNPA2B1-knockdown induced PTEN upregulation and PI3K/AKT signaling inactivation in NSCLC cells. Our findings suggested that HNRNPA2B1 acted by m^6^A-dependent modification of lncRNA MEG3, which acted as a sponge of miR-21-5p to regulate PTEN/PI3K/AKT signaling, contributing to NSCLC progression.

However, our study has some limitations. Firstly, a larger sample size is needed to further validate the prognostic significance of HNRNPA2B1 in patients with NSCLC. Secondly, regarding the upstream modification, it has been reported that SUMOylation of HNRNPA2B1 regulates replication protein A dynamics during genotoxic stress responses [44]. Whether SUMOylation of HNRNPA2B1 modifies NSCLC progression need be explored. Thirdly, whether HNRNPA2B1 mediates m^6^A-dependent modification of mRNAs or circRNAs in NSCLC need be further studied. In addition, mitochondrial biogenesis is necessary for efficient cell function and plays a key role in cancer [45, 46]. Whether HNRNPA2B1 acts by targeting mitochondria in NSCLC need be investigated in the future.

In conclusion, our findings demonstrate that upregulation of HNRNPA2B1 is associated with distant metastasis and poor survival, representing an independent prognostic factor in patients with NSCLC. HNRNPA2B1-mediated m^6^A modification of lncRNA MEG3 promotes tumorigenesis and metastasis of NSCLC by regulating miR-21-5p/PTEN axis and may offer a new epigenetic regulatory mechanism and a potential therapeutic target for NSCLC.

## Supplementary Information

**Additional file 1: Figure S1.** RT-qPCR analysis of the transfection efficiency of si-MEG3 in 95D and H1299 cells.

**Additional file 2: Figure S2.** GES14814 and GES37745 analysis of the association of lncRNA MEG3 expression with the prognosis in patients with NSCLC.

**Additional file 3: Figure S3.** Schematic representation of potential binding sites between miR-21-5p and MEG3.

**Additional file 4: Figure S4.** RT-qPCR analysis of the transfection efficiency of miR-21-5p mimics or inhibitor in 95D and H1299 cells.

**Additional file 5: Table S1.** The sequences of the primers. **Table S2.** The association of HNRNPA2B1 expression with clinicopathological characteristics of LAC patients. **Table S3.** Cox regression analysis of HNRNPA2B1 expression as survival predictor. **Table S4.** The correlation of MEG3 expression with clinicopathologic characteristics of LAC patients. **Table S5.** The association of miR-21-5p expression with clinicopathological characteristics of LAC patients. **Table S6.** Cox regression analysis of miR-21-5p expression as survival predictor.

## Data Availability

All data generated or analysed during this study are included in this published article and its additional files.
