# Supplementary figures and images for: HNRNPA2B1-mediated m6A modification of lncRNA MEG3 facilitates tumorigenesis and metastasis of non-small cell lung cancer by regulating miR-21-5p/PTEN axis
Source: J Transl Med. 2023 Jun 12;21:382. doi: 10.1186/s12967-023-04190-8 (PMC10258935; doi:10.1186/s12967-023-04190-8)

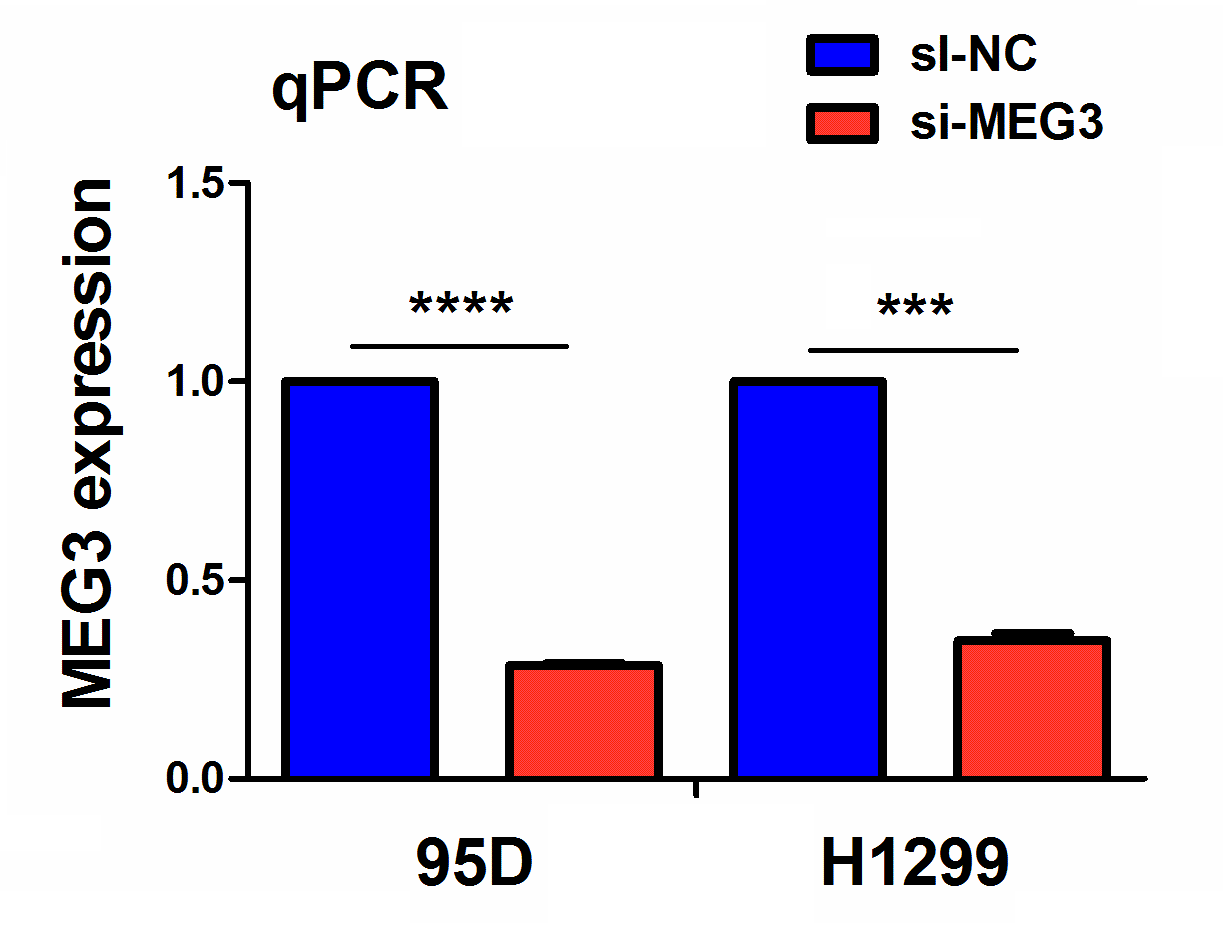

Supplement: Supplementary file 1 — Additional file 1: Figure S1. RT-qPCR analysis of the transfection efficiency of si-MEG3 in 95D and H1299 cells. [file 12967_2023_4190_MOESM1_ESM.tif]

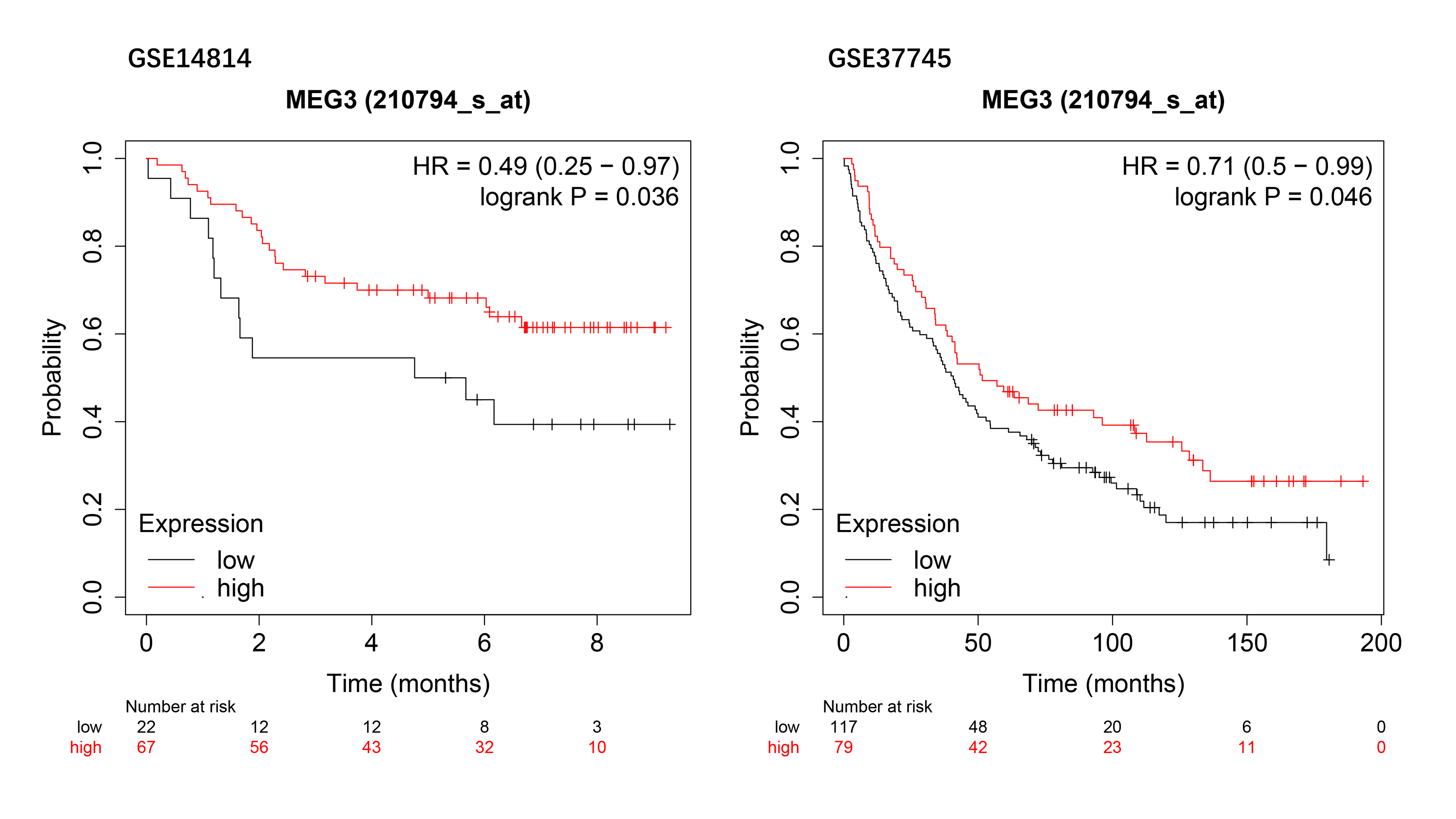

Supplement: Supplementary file 2 — Additional file 2: Figure S2. GES14814 and GES37745 analysis of the association of lncRNA MEG3 expression with the prognosis in patients with NSCLC. [file 12967_2023_4190_MOESM2_ESM.tif]

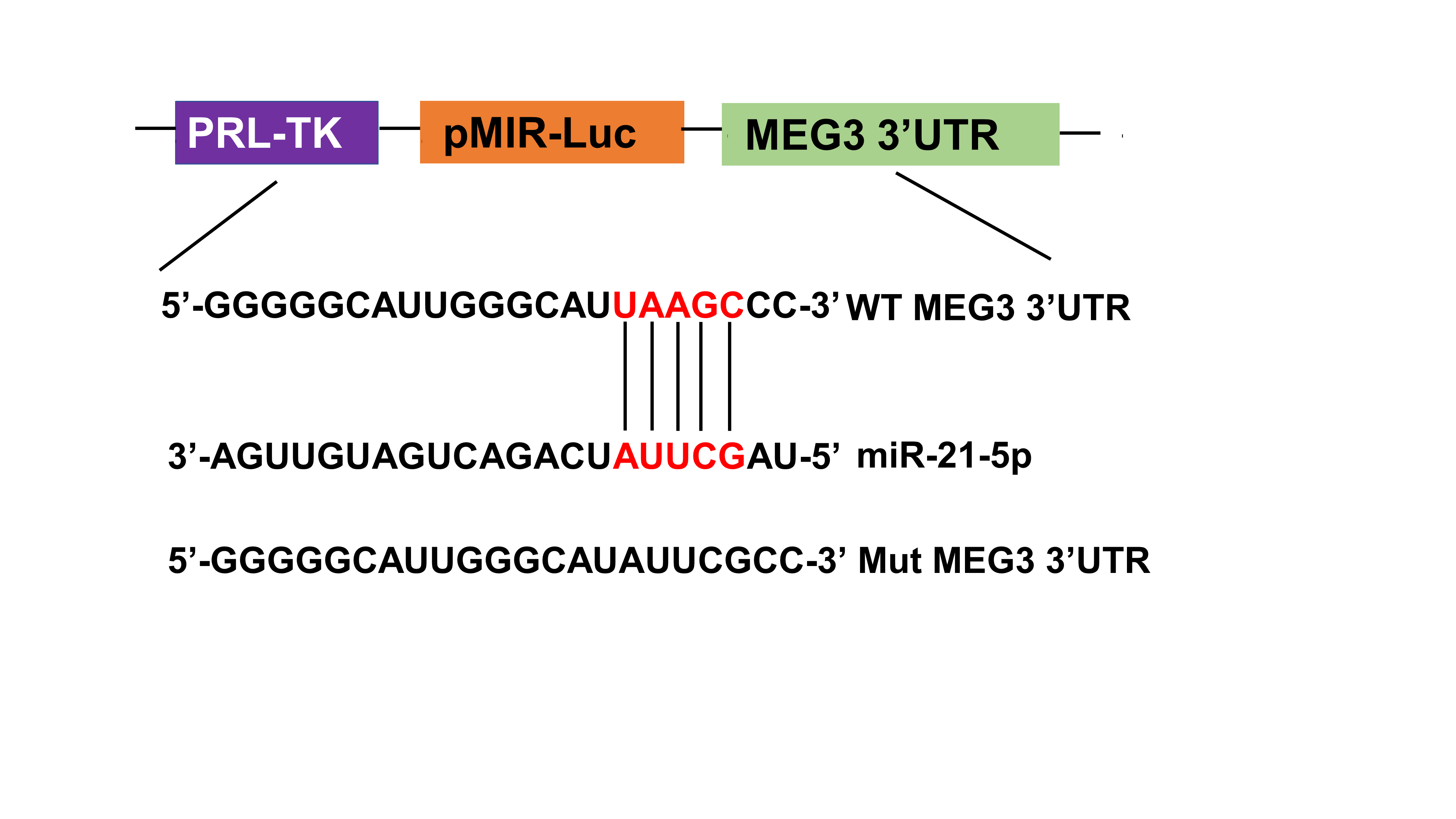

Supplement: Supplementary file 3 — Additional file 3: Figure S3. Schematic representation of potential binding sites between miR-21-5p and MEG3. [file 12967_2023_4190_MOESM3_ESM.tif]

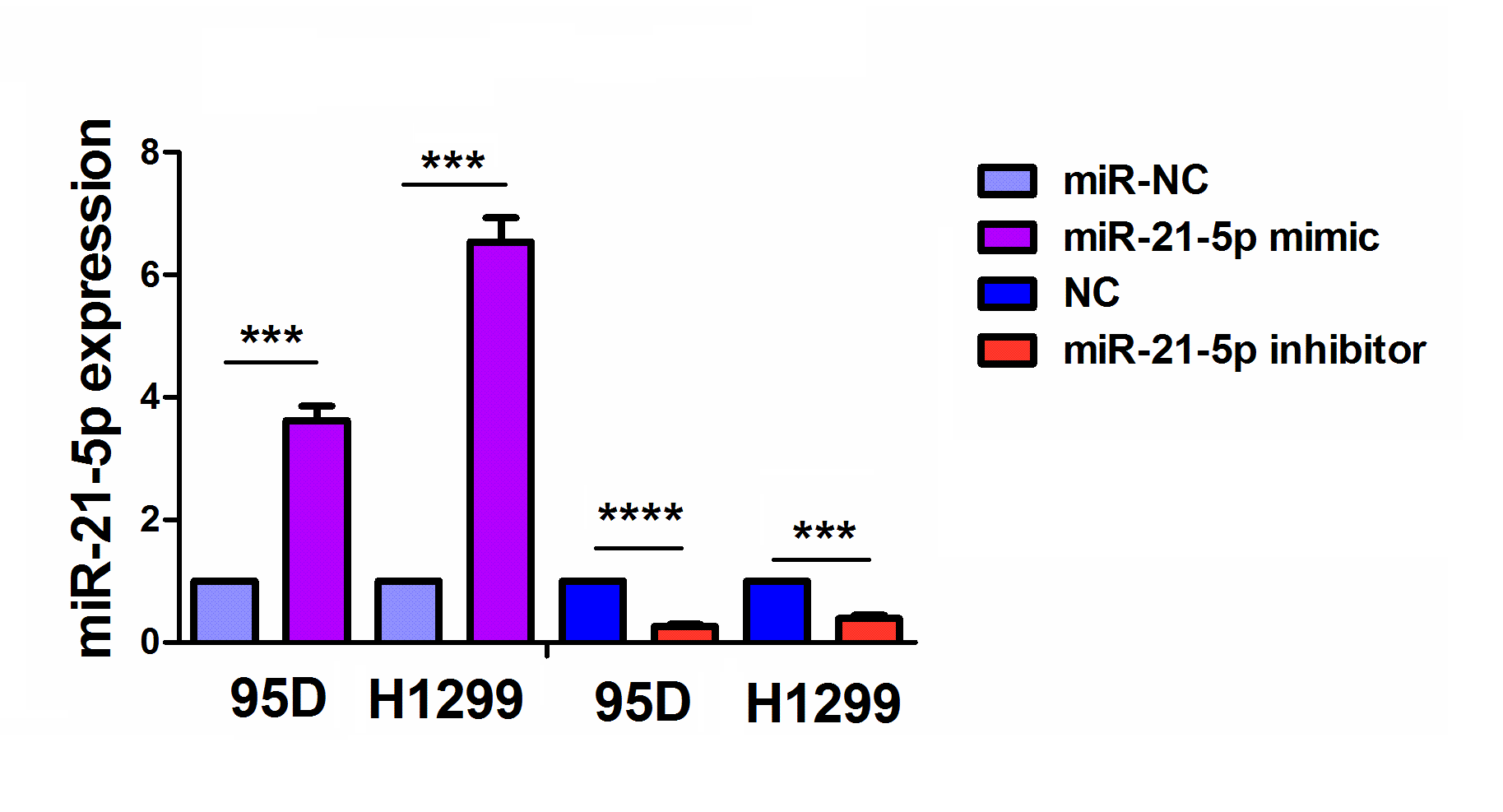

Supplement: Supplementary file 4 — Additional file 4: Figure S4. RT-qPCR analysis of the transfection efficiency of miR-21-5p mimics or inhibitor in 95D and H1299 cells. [file 12967_2023_4190_MOESM4_ESM.tif]
