# Supplementary material for: HNRNPA2B1-mediated m6A modification of lncRNA MEG3 facilitates tumorigenesis and metastasis of non-small cell lung cancer by regulating miR-21-5p/PTEN axis
Source: J Transl Med. 2023 Jun 12;21:382. doi: 10.1186/s12967-023-04190-8 (PMC10258935; doi:10.1186/s12967-023-04190-8)
Supplement: Supplementary file 5 — Additional file 5: Table S1. The sequences of the primers. Table S2. The association of HNRNPA2B1 expression with clinicopathological characteristics of LAC patients. Table S3. Cox regression analysis of HNRNPA2B1 expression as survival predictor. Table S4. The correlation of MEG3 expression with clinicopathologic characteristics of LAC patients. Table S5. The association of miR-21-5p expression with clinicopathological characteristics of LAC patients. Table S6. Cox regression analysis of miR-21-5p expression as survival predictor. [file 12967_2023_4190_MOESM5_ESM.docx]

**Additional Figure legends**

**Additional Figure S1:** RT-qPCR analysis of the transfection efficiency of si-MEG3 in 95D and H1299 cells.

**Additional Figure S2:** GES14814 and GES37745 analysis of the association of lncRNA MEG3 expression with the prognosis in patients with NSCLC.

**Additional Figure S3:** Schematic representation of potential binding sites between miR-21-5p and MEG3.

**Additional Figure S4:** RT-qPCR analysis of the transfection efficiency of miR-21-5p mimics or inhibitor in 95D and H1299 cells.

**Additional Tables**

**Table S1.** The sequences of the primers

| Gene | Primer | Sequence (5'-3') |
| --- | --- | --- |
| U6 | Forward | CGCTTCGGCAGCACATATAC |
|  | Reverse | AAATATGGAACGCTTCACGA |
| homo GAPDH | Forward | TCAAGAAGGTGGTGAAGCAGG |
|  | Reverse | TCAAAGGTGGAGGAGTGGGT |
| Homo m^6^A | Forward | CTGAGCTGAACATGACCTTCC |
|  | Reverse | TGGAGCGAGTAGAGTGGATGT |
| HNRHPA2B1 | Forward | TAATGAGGGATCCTGCAAGC |
|  | Reverse | CAGTTACATGAGCCCCTGGT |
| LncRNA MEG3 | Forward | ACCAACATACAAAGCAGCCACT |
|  | Reverse | GGGTGATGACAGAGTCAGTCGA |
| hsa-miR-21-5p | Loop | GTCGTATCCAGTGCAGGGTCCGAGGTATTCGCACTGGATACGACTCAACATC |
|  | Forward | TGCGCTAGCTTATCAGACTGAT |
|  | Reverse | CCAGTGCAGGGTCCGAGGTATT |
| PTEN | Forward | ACTATTCCCAGTCAGAGGCG |
|  | Reverse | GAACTTGTCTTCCCGTCGTG |

**Table S2** The association of HNRNPA2B1 expression with clinicopathological

characteristics of LAC patients

| Variables | Cases  (n) | HNRNPA2B1 | | *P* value |
| --- | --- | --- | --- | --- |
|  |  | High | Low |  |
| Total | 407 | 121 | 286 |  |
| *Age (years)* |  |  |  |  |
| ≥60 | 293 | 87 | 206 |  |
| <60 | 114 | 34 | 80 | 1.000 |
| *Gender* |  |  |  |  |
| Male | 184 | 67 | 117 |  |
| Female | 223 | 54 | 169 | 0.009 |
| *Pathological stage* |  |  |  |  |
| Ⅰ/Ⅱ | 327 | 93 | 234 |  |
| Ⅲ/Ⅳ | 80 | 28 | 52 | 0.276 |
| *T stage* |  |  |  |  |
| T1/T2 | 358 | 105 | 253 |  |
| T3/T4 | 49 | 16 | 33 | 0.621 |
| *N stage* |  |  |  |  |
| Negative | 269 | 81 | 188 |  |
| Positive | 138 | 40 | 98 | 0.909 |
| *M stage* |  |  |  |  |
| Negative | 260 | 64 | 196 |  |
| Positive | 147 | 57 | 90 | 0.003 |

**Table S3** Cox regression analysis of HNRNPA2B1 expression as survival predictor

| Variables | Univariate Cox regression analysis | |  | Multivariate Cox regression analysis | |
| --- | --- | --- | --- | --- | --- |
|  | RR (95% CI) | *P* value |  | RR (95% CI) | *P* value |
| *Age (years)* |  |  |  |  |  |
| <60 vs. ≥60 | 1.006 (0.691 to 1.466) | 0.974 |  | NA | NA |
| *Gender* |  |  |  |  |  |
| Male vs. Female | 1.042 (0.741 to 1.464) | 0.814 |  | NA | NA |
| *Pathological stage* |  |  |  |  |  |
| Ⅲ/Ⅳ vs.Ⅰ/Ⅱ | 2.399 (1.669 to 3.447) | <0.0001 |  | 1.380 (0.863 to 2.207) | 0.179 |
| *T stage* |  |  |  |  |  |
| T3+T4 vs. T1+T2 | 2.171 (1.378 to 3.420) | 0.001 |  | 1.555 (0.949 to 2.549) | 0.080 |
| *N staging* |  |  |  |  |  |
| Positive vs. Negative | 2.236 (1.592 to 3.139) | <0.0001 |  | 1.861 (1.232 to 2.809) | 0.003 |
| *M stage* |  |  |  |  |  |
| Positive vs. Negative | 0.969 (0.675 to 1.392) | 0.866 |  | NA | NA |
| *HNRNPA2B1 expression* |  |  |  |  |  |
| High vs. Low | 1.906 (1.346 to 2.699) | <0.0001 |  | 1.804 (1.268 to 2.568) | 0.001 |

NA: not analyzed

**Table S4** The correlation of MEG3 expression with clinicopathologic

characteristics of LAC patients

| Variables | Cases  (n) | MEG3 | | *P* value |
| --- | --- | --- | --- | --- |
|  |  | High | Low |  |
| Total | 479 | 312 | 167 |  |
| *Age (years)* |  |  |  |  |
| ≥60 | 348 | 224 | 124 |  |
| <60 | 131 | 88 | 43 | 0.592 |
| *Gender* |  |  |  |  |
| Male | 220 | 134 | 86 |  |
| Female | 259 | 178 | 81 | 0.083 |
| *Pathological stage* |  |  |  |  |
| Ⅰ/Ⅱ | 378 | 255 | 123 |  |
| Ⅲ/Ⅳ | 101 | 57 | 44 | 0.046 |
| *T stage* |  |  |  |  |
| T1/T2 | 416 | 277 | 139 |  |
| T3/T4 | 63 | 35 | 28 | 0.091 |
| *N stage* |  |  |  |  |
| Negative | 312 | 219 | 93 |  |
| Positive | 167 | 93 | 74 | 0.002 |
| *M stage* |  |  |  |  |
| Negative | 317 | 198 | 119 |  |
| Positive | 162 | 114 | 48 | 0.105 |

**Table S5** The association of miR-21-5p expression with clinicopathological

characteristics of LAC patients

| Variables | Cases  (n) | miR-21-5p | | *P* value |
| --- | --- | --- | --- | --- |
|  |  | High | Low |  |
| Total | 419 | 252 | 167 |  |
| *Age (years)* |  |  |  |  |
| ≥60 | 302 | 176 | 126 |  |
| <60 | 117 | 76 | 41 | 0.223 |
| *Gender* |  |  |  |  |
| Male | 194 | 115 | 79 |  |
| Female | 225 | 137 | 88 | 0.765 |
| *Pathological stage* |  |  |  |  |
| Ⅰ/Ⅱ | 332 | 200 | 132 |  |
| Ⅲ/Ⅳ | 87 | 52 | 35 | 1.000 |
| *T stage* |  |  |  |  |
| T1/T2 | 363 | 222 | 141 |  |
| T3/T4 | 56 | 30 | 26 | 0.306 |
| *N stage* |  |  |  |  |
| Negative | 277 | 154 | 123 |  |
| Positive | 142 | 98 | 44 | 0.008 |
| *M stage* |  |  |  |  |
| Negative | 261 | 166 | 95 |  |
| Positive | 158 | 86 | 72 | 0.065 |

**Table S6** Cox regression analysis of miR-21-5p expression as survival predictor

| Variables | Univariate Cox regression analysis | |  | Multivariate Cox regression analysis | |
| --- | --- | --- | --- | --- | --- |
|  | RR (95% CI) | *P* value |  | RR (95% CI) | *P* value |
| *Age (years)* |  |  |  |  |  |
| <60 vs. ≥60 | 1.013 (0.727 to 1.411) | 0.940 |  | NA | NA |
| *Gender* |  |  |  |  |  |
| Male vs. Female | 1.084 (0.807 to 1.455) | 0.593 |  | NA | NA |
| *Pathological stage* |  |  |  |  |  |
| Ⅲ/Ⅳ vs.Ⅰ/Ⅱ | 2.494 (1.825 to 3.410) | <0.001 |  | 1.558 (1.006 to 2.411) | 0.047 |
| *T stage* |  |  |  |  |  |
| T3+T4 vs. T1+T2 | 2.288 (1.574 to 3.324) | <0.001 |  | 1.603 (1.023 to 2.513) | 0.040 |
| *N staging* |  |  |  |  |  |
| Positive vs. Negative | 2.378 (1.769 to 3.197) | <0.001 |  | 1.725 (1.159 to 2.569) | 0.007 |
| *M stage* |  |  |  |  |  |
| Positive vs. Negative | 0.985 (0.715 to 1.357) | 0.927 |  | NA | NA |
| *miR-21-5p expression* |  |  |  |  |  |
| High vs. Low | 1.601 (1.135 to 2.258) | 0.007 |  | 1.385 (0.967 to 1.982) | 0.075 |

NA: not analyzed
